# 2-(4-Chloro-3,3,7-trimethyl-2,3-dihydro-1*H*-indol-2-yl­idene)-2-cyano­acetamide

**DOI:** 10.1107/S1600536811053918

**Published:** 2011-12-23

**Authors:** Madeleine Helliwell, Mehdi M. Baradarani, Maryam Alyari, Arash Afghan, John A. Joule

**Affiliations:** a The School of Chemistry, The University of Manchester, Manchester M13 9PL, England; b Department of Chemistry, Faculty of Science, University of Urmia, Urmia 57153-165, Iran; c Department of Chemical Engineering, University of Urmia, Urmia 57153-165, Iran

## Abstract

Reaction of 2-(4-chloro-3,3,7-trimethyl-2,3-dihydro-1*H*-indol-2-yl­idene)propane­dial with hydroxyl­amine gives the title compound, C_14_H_14_ClN_3_O, in which the ring N atom is essentially planar [sum of angles around the ring N atom = 361°], indicating conjugation with the 2-cyano­acryl­amide unit. The orientation of the acetamide group arises from intra­molecular hydrogen bonding between the indole N—H and carbonyl groups. In the crystal, inversion-related acetamide groups form N—H⋯O hydrogen-bonded dimers in graph-set *R*_2_^2^(8) motifs, whilst dimers are also formed by pairs of amine–nitrile N—H⋯N hydrogen bonds in *R*_2_^2^(12) motifs. These inter­actions together generate ribbons that propagate along the *b*-axis direction.

## Related literature

For background information on the chemistry of related compounds, see: Baradarani *et al.* (2006 ▶); Rashidi *et al.* (2009 ▶, 2011 ▶). For related structures, see: Helliwell *et al.* (2010 ▶, 2012 ▶). For graph-set notation, see: Etter *et al.* (1990 ▶).
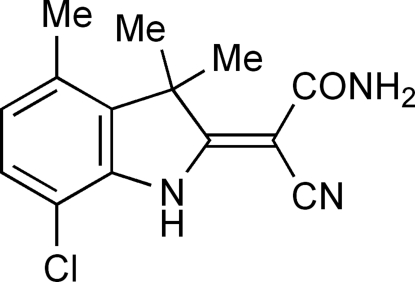


## Experimental

### 

#### Crystal data

C_14_H_14_ClN_3_O
*M* *_r_* = 275.73
Triclinic, 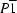

*a* = 8.226 (2) Å
*b* = 9.282 (3) Å
*c* = 9.744 (3) Å
α = 92.124 (5)°
β = 104.766 (5)°
γ = 105.294 (4)°
*V* = 689.5 (3) Å^3^
*Z* = 2
Mo *K*α radiation
μ = 0.27 mm^−1^
*T* = 100 K
0.58 × 0.22 × 0.10 mm

#### Data collection

Bruker SMART CCD area-detector diffractometer
3384 measured reflections
2389 independent reflections
1549 reflections with *I* > 2σ(*I*)
*R*_int_ = 0.065

#### Refinement

*R*[*F* ^2^ > 2σ(*F* ^2^)] = 0.065
*wR*(*F* ^2^) = 0.132
*S* = 1.01
2389 reflections
187 parameters
H atoms treated by a mixture of independent and constrained refinement
Δρ_max_ = 0.45 e Å^−3^
Δρ_min_ = −0.38 e Å^−3^

### 

Data collection: *SMART* (Bruker, 2001 ▶); cell refinement: *SAINT* (Bruker, 2002 ▶); data reduction: *SAINT*; program(s) used to solve structure: *SHELXS97* (Sheldrick, 2008 ▶); program(s) used to refine structure: *SHELXL97* (Sheldrick, 2008 ▶); molecular graphics: *XP* in *SHELXTL* (Sheldrick, 2008 ▶); software used to prepare material for publication: *SHELXTL*.

## Supplementary Material

Crystal structure: contains datablock(s) global, I. DOI: 10.1107/S1600536811053918/pk2373sup1.cif

Structure factors: contains datablock(s) I. DOI: 10.1107/S1600536811053918/pk2373Isup2.hkl

Supplementary material file. DOI: 10.1107/S1600536811053918/pk2373Isup3.cml

Additional supplementary materials:  crystallographic information; 3D view; checkCIF report

## Figures and Tables

**Table 1:** Hydrogen-bond geometry (Å, °)

*D*—H⋯*A*	*D*—H	H⋯*A*	*D*⋯*A*	*D*—H⋯*A*
N1—H1*N*⋯O1	0.93 (5)	1.89 (5)	2.610 (4)	132 (4)
N3—H3*M*⋯O1^i^	0.83 (5)	2.11 (5)	2.931 (5)	176 (5)
N3—H3*N*⋯N2^ii^	0.85 (4)	2.24 (4)	3.065 (5)	163 (3)

Symmetry codes: (i) 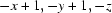; (ii) 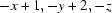.

**Fig. 1.**
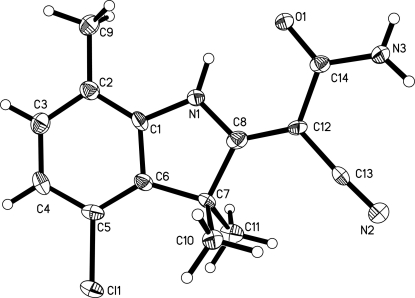
A plot of the title compound with ellipsoids drawn at the 50% probability level.

**Fig. 2.**
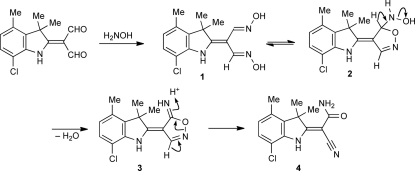
Synthesis of 2-(4-chloro-1,3-dihydro-3,3,7-trimethyl -2H-indol-2-ylidene)-2-cyanoacetamide

**Fig. 3.**
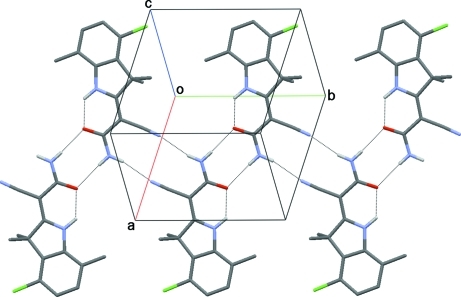
Packing arrangement of the title compound showing the H bonding which links the molecules into chains parallel to b. Only those H atoms that are involved in H bonding are shown for clarity.

## supplementary crystallographic information

### Comment

We showed that the interaction of 2,3,3-trimethyl-3*H*-indoles with the Vilsmeier reagent produces (1,3-dihydro-3,3-dimethyl-2*H*-indol-2-ylidene)propanedials (Baradarani *et al.*, 2006). 2,3,3-Trimethyl-2*H*-pyrrolo[2,3-*f*]quinoline, 2,3,3-trimethyl-3*H*-pyrrolo[3,2-*h*]quinoline (Rashidi *et al.*, 2009), 2,2',3,3,3',3'-hexamethyl-3*H*,3'*H*-5,5'-biindole and 2,3,3,7,8,8-hexamethyl-3*H*,8*H*-indolo[7,6-*g*]indole (Rashidi *et al.*, 2011) behave analogously. The (1,3-dihydroindol-2-ylidene)propanedials were shown to react with arylhydrazines (or hydrazine) to produce 3,3-dimethyl-2-[1-aryl-1*H*-pyrazol-4-yl]-3*H*-indoles (Baradarani *et al.*, 2006. Rashidi *et al.*, 2009. Helliwell *et al.* 2010. Rashidi *et al.*, 2011).

In anticipation that the (1,3-dihydroindol-2-ylidene)propanedials would react with hydroxylamine to produce isoxazol-4-yl-3*H*-indoles, 2-(4-chloro-1,3-dihydro-3,3,7-trimethyl-2*H*-indol-2-ylidene)propanedial was treated with hydroxylamine in refluxing ethanol. The unexpected product of the reaction was 2-(4-chloro-1,3-dihydro-3,3,7-trimethyl-2*H*-indol-2-ylidene)-2- cyanoacetamide as shown by this X-ray analysis (Fig. 1).

We interpret this transformation as involving firstly formation of the diooxime 1 which cyclizes to generate aminal 2, loss of water would then produce imine 3, proton-induced fragmentation of which (arrows on 3) would then lead to the product 4 (Fig. 2).

The sum of the angles of the bonds at the ring nitrogen in 4 is 361 (4)° showing the extensive conjugation of the nitrogen with the 2-cyanoacrylamide subunit. The geometry of the double bond linking the heterocyclic and cyanoacetamide subunits is *E*.

The orientation of the acetamide group arises from intramolecular H bonding between the indole N—H and the carbonyl group. The inversion related acetamide groups form N—H···O hydrogen bonded dimers in graph-set *R*^2^_2_(8) motifs (Etter *et al.*, 1990), while dimers are also formed by pairs of *N*—*H*(amine)···*N*(nitrile) functionalities in *R*^2^_2_(12) motifs.  These interactions together generate ribbons that propagate along the *b* axis direction (Fig. 3).

### Experimental

A mixture of 2-(4-chloro-1,3-dihydro-3,3,7-trimethyl-2*H*-indol-2-ylidene)propanedial (100 mg, 0.38 mmol) and hydroxylamine hydrochloride (52 mg, 0.76 mmol) in absolute EtOH (10 ml) was heated with stirring at reflux for 7 h. After complete conversion, the reaction mixture was cooled and concentrated, and the resulting crystals were collected by filtration and recrystallized from EtOH to give the 2-(4-chloro-1,3-dihydro-3,3,7-trimethyl-2*H*-indol-2-ylidene)-2-cyanoacrylamide (90 mg, 86%), 522–524 K, FT—IR (KBr) ν_max_ 3385, 3289, 3185, 2986, 2939, 2199, 1668, 1608, 1563, 1415, 1329, 909, 788 cm^-1^, ^1^H NMR (CDCl_3_) δ 1.84 (s, 6H, 2CH_3_), 2.30 (s, 3H, CH_3_), 5.20–6.50 (bs, 2H, NH_2_), 6.93 (d, *J* = 8.1 Hz, 1H, ArH), 7.00 (d, *J* = 8.1 Hz, 1H, ArH), 11.85 (bs, 1H, NH), ^13^C NMR (CDCl_3_) δ 15.9, 21.0, 51.6, 118.9, 118.9, 124.9, 127.4, 130.8, 132.5, 141.0, 169.6,177.4.

### Refinement

H atoms bonded to C were included in calculated positions using the riding method, with C—H distances of 0.98 Å and *U*_iso_(H) = 1.5*U*_eq_(C) for methyl H atoms and C—H distances of 0.95 Å and *U*_iso_(H) = 1.2*U*_eq_(C) for the aromatic H atoms. Those bonded to N were found by difference Fourier methods and refined isotropically with the N—H distances ranging from to 0.83 (5) to 0.93 (5) Å.

### Crystal data

**Table:**

C_14_H_14_ClN_3_O	*Z* = 2
*M**_r_* = 275.73	*F*(000) = 288
Triclinic, *P*1	*D*_x_ = 1.328 Mg m^−^^3^
Hall symbol: -P 1	Mo *K*α radiation, λ = 0.71073 Å
*a* = 8.226 (2) Å	Cell parameters from 735 reflections
*b* = 9.282 (3) Å	θ = 2.7–25.8°
*c* = 9.744 (3) Å	µ = 0.27 mm^−^^1^
α = 92.124 (5)°	*T* = 100 K
β = 104.766 (5)°	Plate, colourless
γ = 105.294 (4)°	0.58 × 0.22 × 0.10 mm
*V* = 689.5 (3) Å^3^	

### Data collection

**Table:**

Bruker SMART CCD area-detector diffractometer	1549 reflections with *I* > 2σ(*I*)
Radiation source: fine-focus sealed tube	*R*_int_ = 0.065
graphite	θ_max_ = 25.0°, θ_min_ = 2.2°
φ and ω scans	*h* = −9→9
3384 measured reflections	*k* = −10→11
2389 independent reflections	*l* = −10→11

### Refinement

**Table:**

Refinement on *F*^2^	Primary atom site location: structure-invariant direct methods
Least-squares matrix: full	Secondary atom site location: difference Fourier map
*R*[*F*^2^ > 2σ(*F*^2^)] = 0.065	Hydrogen site location: inferred from neighbouring sites
*wR*(*F*^2^) = 0.132	H atoms treated by a mixture of independent and constrained refinement
*S* = 1.01	*w* = 1/[σ^2^(*F*_o_^2^) + (0.0419*P*)^2^] where *P* = (*F*_o_^2^ + 2*F*_c_^2^)/3
2389 reflections	(Δ/σ)_max_ < 0.001
187 parameters	Δρ_max_ = 0.45 e Å^−^^3^
0 restraints	Δρ_min_ = −0.38 e Å^−^^3^

### Special details

**Table:**

Geometry. All e.s.d.'s (except the e.s.d. in the dihedral angle between two l.s. planes) are estimated using the full covariance matrix. The cell e.s.d.'s are taken into account individually in the estimation of e.s.d.'s in distances, angles and torsion angles; correlations between e.s.d.'s in cell parameters are only used when they are defined by crystal symmetry. An approximate (isotropic) treatment of cell e.s.d.'s is used for estimating e.s.d.'s involving l.s. planes.
Refinement. Refinement of *F*^2^ against ALL reflections. The weighted *R*-factor *wR* and goodness of fit *S* are based on *F*^2^, conventional *R*-factors *R* are based on *F*, with *F* set to zero for negative *F*^2^. The threshold expression of *F*^2^ > σ(*F*^2^) is used only for calculating *R*-factors(gt) *etc*. and is not relevant to the choice of reflections for refinement. *R*-factors based on *F*^2^ are statistically about twice as large as those based on *F*, and *R*- factors based on ALL data will be even larger.

### Fractional atomic coordinates and isotropic or equivalent isotropic displacement parameters (Å^2^)

**Table:**

	*x*	*y*	*z*	*U*_iso_*/*U*_eq_	
Cl1	−0.29616 (14)	0.79125 (12)	0.38519 (11)	0.0274 (3)	
O1	0.3245 (3)	0.4944 (3)	0.0925 (3)	0.0224 (7)	
N1	0.0818 (4)	0.5294 (4)	0.2080 (3)	0.0190 (8)	
H1N	0.140 (6)	0.464 (6)	0.181 (5)	0.064 (17)*	
N2	0.3940 (5)	1.0145 (4)	0.1147 (4)	0.0306 (9)	
N3	0.4858 (5)	0.6920 (5)	0.0127 (4)	0.0225 (8)	
H3N	0.517 (5)	0.783 (5)	−0.006 (4)	0.022 (12)*	
H3M	0.539 (7)	0.637 (6)	−0.013 (5)	0.061 (18)*	
C1	−0.0620 (5)	0.4992 (4)	0.2658 (4)	0.0182 (9)	
C2	−0.1558 (5)	0.3606 (5)	0.2912 (4)	0.0207 (9)	
C3	−0.2983 (5)	0.3602 (5)	0.3433 (4)	0.0244 (10)	
H3	−0.3693	0.2673	0.3598	0.029*	
C4	−0.3399 (5)	0.4911 (5)	0.3718 (4)	0.0241 (10)	
H4	−0.4370	0.4878	0.4087	0.029*	
C5	−0.2383 (5)	0.6280 (5)	0.3461 (4)	0.0218 (10)	
C6	−0.0984 (5)	0.6349 (4)	0.2903 (4)	0.0179 (9)	
C7	0.0298 (5)	0.7621 (4)	0.2452 (4)	0.0174 (9)	
C8	0.1405 (5)	0.6759 (4)	0.1913 (4)	0.0194 (9)	
C9	−0.1084 (5)	0.2188 (4)	0.2602 (5)	0.0271 (10)	
H9A	−0.0865	0.2171	0.1660	0.041*	
H9B	−0.2053	0.1312	0.2613	0.041*	
H9C	−0.0027	0.2161	0.3332	0.041*	
C10	−0.0645 (5)	0.8363 (4)	0.1230 (4)	0.0233 (10)	
H10A	0.0218	0.9169	0.0962	0.035*	
H10B	−0.1481	0.8784	0.1546	0.035*	
H10C	−0.1274	0.7610	0.0403	0.035*	
C11	0.1435 (5)	0.8796 (5)	0.3722 (4)	0.0262 (10)	
H11A	0.2078	0.8307	0.4460	0.039*	
H11B	0.0681	0.9253	0.4117	0.039*	
H11C	0.2268	0.9577	0.3397	0.039*	
C12	0.2765 (5)	0.7319 (4)	0.1318 (4)	0.0183 (9)	
C13	0.3378 (5)	0.8890 (5)	0.1241 (4)	0.0216 (10)	
C14	0.3650 (5)	0.6315 (4)	0.0777 (4)	0.0174 (9)	

### Atomic displacement parameters (Å^2^)

**Table:**

	*U*^11^	*U*^22^	*U*^33^	*U*^12^	*U*^13^	*U*^23^
Cl1	0.0283 (6)	0.0262 (7)	0.0351 (7)	0.0153 (5)	0.0142 (5)	0.0008 (5)
O1	0.0237 (16)	0.0140 (17)	0.0356 (18)	0.0083 (12)	0.0150 (13)	0.0072 (13)
N1	0.0193 (19)	0.015 (2)	0.027 (2)	0.0076 (15)	0.0104 (16)	0.0061 (15)
N2	0.031 (2)	0.019 (2)	0.048 (2)	0.0074 (18)	0.0209 (19)	0.0073 (19)
N3	0.023 (2)	0.015 (2)	0.035 (2)	0.0071 (17)	0.0143 (17)	0.0068 (18)
C1	0.015 (2)	0.024 (2)	0.020 (2)	0.0082 (18)	0.0094 (18)	0.0058 (18)
C2	0.020 (2)	0.022 (2)	0.022 (2)	0.0093 (19)	0.0067 (18)	0.0052 (18)
C3	0.026 (2)	0.022 (2)	0.028 (2)	0.0052 (19)	0.012 (2)	0.0083 (19)
C4	0.017 (2)	0.033 (3)	0.025 (2)	0.007 (2)	0.0097 (19)	0.004 (2)
C5	0.024 (2)	0.021 (2)	0.024 (2)	0.0132 (19)	0.0052 (19)	0.0006 (19)
C6	0.017 (2)	0.020 (2)	0.019 (2)	0.0095 (18)	0.0057 (18)	0.0044 (18)
C7	0.014 (2)	0.018 (2)	0.024 (2)	0.0087 (17)	0.0070 (18)	0.0033 (18)
C8	0.020 (2)	0.017 (2)	0.021 (2)	0.0086 (18)	0.0017 (18)	0.0054 (18)
C9	0.030 (3)	0.018 (2)	0.037 (3)	0.007 (2)	0.015 (2)	0.009 (2)
C10	0.021 (2)	0.019 (2)	0.032 (3)	0.0074 (18)	0.0093 (19)	0.0061 (19)
C11	0.028 (3)	0.026 (3)	0.026 (2)	0.012 (2)	0.0059 (19)	0.000 (2)
C12	0.019 (2)	0.013 (2)	0.026 (2)	0.0092 (17)	0.0069 (18)	0.0057 (18)
C13	0.017 (2)	0.022 (3)	0.031 (2)	0.0081 (19)	0.0137 (19)	0.004 (2)
C14	0.016 (2)	0.016 (2)	0.021 (2)	0.0073 (17)	0.0013 (18)	0.0027 (18)

### Geometric parameters (Å, °)

**Table:**

Cl1—C5	1.760 (4)	C5—C6	1.381 (5)
O1—C14	1.251 (4)	C6—C7	1.523 (5)
N1—C8	1.349 (5)	C7—C8	1.531 (5)
N1—C1	1.404 (4)	C7—C11	1.538 (5)
N1—H1N	0.93 (5)	C7—C10	1.538 (5)
N2—C13	1.151 (5)	C8—C12	1.380 (5)
N3—C14	1.326 (5)	C9—H9A	0.9800
N3—H3N	0.85 (4)	C9—H9B	0.9800
N3—H3M	0.83 (5)	C9—H9C	0.9800
C1—C2	1.381 (5)	C10—H10A	0.9800
C1—C6	1.395 (5)	C10—H10B	0.9800
C2—C3	1.391 (5)	C10—H10C	0.9800
C2—C9	1.509 (5)	C11—H11A	0.9800
C3—C4	1.383 (5)	C11—H11B	0.9800
C3—H3	0.9500	C11—H11C	0.9800
C4—C5	1.395 (5)	C12—C13	1.424 (5)
C4—H4	0.9500	C12—C14	1.481 (5)
			
C8—N1—C1	112.7 (3)	C11—C7—C10	111.0 (3)
C8—N1—H1N	118 (3)	N1—C8—C12	123.4 (3)
C1—N1—H1N	130 (3)	N1—C8—C7	108.8 (3)
C14—N3—H3N	128 (3)	C12—C8—C7	127.8 (4)
C14—N3—H3M	118 (3)	C2—C9—H9A	109.5
H3N—N3—H3M	114 (4)	C2—C9—H9B	109.5
C2—C1—C6	125.2 (3)	H9A—C9—H9B	109.5
C2—C1—N1	127.0 (3)	C2—C9—H9C	109.5
C6—C1—N1	107.8 (3)	H9A—C9—H9C	109.5
C1—C2—C3	115.8 (4)	H9B—C9—H9C	109.5
C1—C2—C9	121.7 (3)	C7—C10—H10A	109.5
C3—C2—C9	122.5 (4)	C7—C10—H10B	109.5
C4—C3—C2	121.9 (4)	H10A—C10—H10B	109.5
C4—C3—H3	119.0	C7—C10—H10C	109.5
C2—C3—H3	119.0	H10A—C10—H10C	109.5
C3—C4—C5	119.6 (3)	H10B—C10—H10C	109.5
C3—C4—H4	120.2	C7—C11—H11A	109.5
C5—C4—H4	120.2	C7—C11—H11B	109.5
C6—C5—C4	121.1 (4)	H11A—C11—H11B	109.5
C6—C5—Cl1	121.2 (3)	C7—C11—H11C	109.5
C4—C5—Cl1	117.7 (3)	H11A—C11—H11C	109.5
C5—C6—C1	116.4 (4)	H11B—C11—H11C	109.5
C5—C6—C7	133.7 (4)	C8—C12—C13	120.4 (3)
C1—C6—C7	109.9 (3)	C8—C12—C14	121.2 (3)
C6—C7—C8	100.8 (3)	C13—C12—C14	118.4 (3)
C6—C7—C11	111.8 (3)	N2—C13—C12	176.3 (4)
C8—C7—C11	110.9 (3)	O1—C14—N3	122.0 (4)
C6—C7—C10	111.6 (3)	O1—C14—C12	120.4 (3)
C8—C7—C10	110.2 (3)	N3—C14—C12	117.6 (4)
			
C8—N1—C1—C2	177.6 (4)	C5—C6—C7—C11	63.5 (6)
C8—N1—C1—C6	−0.8 (4)	C1—C6—C7—C11	−117.4 (4)
C6—C1—C2—C3	1.0 (6)	C5—C6—C7—C10	−61.6 (6)
N1—C1—C2—C3	−177.1 (4)	C1—C6—C7—C10	117.5 (4)
C6—C1—C2—C9	179.4 (4)	C1—N1—C8—C12	−177.6 (4)
N1—C1—C2—C9	1.2 (6)	C1—N1—C8—C7	1.1 (4)
C1—C2—C3—C4	−1.9 (6)	C6—C7—C8—N1	−1.0 (4)
C9—C2—C3—C4	179.7 (4)	C11—C7—C8—N1	117.6 (4)
C2—C3—C4—C5	0.9 (6)	C10—C7—C8—N1	−119.0 (4)
C3—C4—C5—C6	1.1 (6)	C6—C7—C8—C12	177.7 (4)
C3—C4—C5—Cl1	−179.6 (3)	C11—C7—C8—C12	−63.7 (5)
C4—C5—C6—C1	−2.0 (6)	C10—C7—C8—C12	59.7 (5)
Cl1—C5—C6—C1	178.8 (3)	N1—C8—C12—C13	−176.7 (4)
C4—C5—C6—C7	177.0 (4)	C7—C8—C12—C13	4.7 (6)
Cl1—C5—C6—C7	−2.2 (6)	N1—C8—C12—C14	1.8 (6)
C2—C1—C6—C5	0.9 (6)	C7—C8—C12—C14	−176.7 (4)
N1—C1—C6—C5	179.3 (3)	C8—C12—C14—O1	−4.2 (6)
C2—C1—C6—C7	−178.3 (4)	C13—C12—C14—O1	174.4 (4)
N1—C1—C6—C7	0.1 (4)	C8—C12—C14—N3	175.2 (4)
C5—C6—C7—C8	−178.5 (4)	C13—C12—C14—N3	−6.2 (6)
C1—C6—C7—C8	0.5 (4)		

### Hydrogen-bond geometry (Å, °)

**Table:**

*D*—H···*A*	*D*—H	H···*A*	*D*···*A*	*D*—H···*A*
N1—H1N···O1	0.93 (5)	1.89 (5)	2.610 (4)	132 (4)
N3—H3M···O1^i^	0.83 (5)	2.11 (5)	2.931 (5)	176 (5)
N3—H3N···N2^ii^	0.85 (4)	2.24 (4)	3.065 (5)	163 (3)

Symmetry codes: (i) −*x*+1, −*y*+1, −*z*; (ii) −*x*+1, −*y*+2, −*z*.

## References

[bb1] Baradarani, M. M., Afghan, A., Zebarjadi, F., Hasanzadeh, K. & Joule, J. A. (2006). *J. Heterocycl. Chem.* **43**, 1591–1596.

[bb2] Bruker (2001). *SMART and* *SADABS* Bruker AXS Inc., Madison, Wisconsin, USA.

[bb3] Bruker (2002). *SAINT* Bruker AXS Inc., Madison, Wisconsin, USA.

[bb4] Etter, M. C., MacDonald, J. C. & Bernstein, J. (1990). *Acta Cryst.* B**46**, 256–262. 10.1107/s0108768189012929 2344397

[bb5] Helliwell, M., Afghan, A., Keshvari, F., Baradarani, M. M. & Joule, J. A. (2010). *Acta Cryst.* E**66**, o112. 10.1107/S1600536809052581 PMC2980154 21580001

[bb9] Helliwell, M., Baradarani, M. M., Mohammadnejadaghdam, R., Afghan, A., & Joule, J. A. (2012). *Acta Cryst.* E**68**, o233. 10.1107/S1600536811053906 PMC3254565 22259515

[bb6] Rashidi, A., Afghan, A., Baradarani, M. M. & Joule, J. A. (2009). *J. Heterocycl. Chem.* **46**, 428–431.

[bb7] Rashidi, A., Baradarani, M. M. & Joule, J. A. (2011). *Arkivoc*, **ii**, 252–259.

[bb8] Sheldrick, G. M. (2008). *Acta Cryst.* A**64**, 112–122. 10.1107/S0108767307043930 18156677

